# Healing Delayed, Healing Restored: Role of Vitamin D in Periodontal Surgery With a 10-Month Follow-Up

**DOI:** 10.7759/cureus.88585

**Published:** 2025-07-23

**Authors:** Mayur Kaushik, Yashica Dhingra, Riya Agarwal, Soamya Gandhi

**Affiliations:** 1 Department of Periodontology and Implantology, Subharti Dental College and Hospital, Swami Vivekanand Subharti University, Meerut, IND; 2 Department of Periodontology, Swami Vivekanand Subharti University, Meerut, IND

**Keywords:** delayed wound healing, electrosurgery, esthetic crown lengthening, esthetic dentistry, vitamin d

## Abstract

Wound healing is a complex and multifaceted process that is vital for restoring tissue integrity after surgical procedures. Vitamin D deficiency has been associated with delayed wound closure, increased inflammation, and impaired epithelialization. This case report aims to explore the relationship between vitamin D deficiency and delayed wound healing following depigmentation, frenectomy, and crown lengthening procedures performed with electrosurgery. This case illustrates the critical but often overlooked role of vitamin D in oral soft tissue repair, even in young and otherwise healthy individuals. Vitamin D deficiency should be considered in the differential diagnosis of delayed oral wound healing, even in young, systemically healthy patients. Early identification and correction can significantly improve outcomes and prevent unnecessary interventions or misdiagnosis. This case highlights the importance of a holistic, systemic approach in managing postoperative complications associated with aesthetic periodontal therapy.

## Introduction

Individuals who experience efficient wound healing differ significantly from those with chronic, non-healing lesions, primarily due to variations in the effectiveness of the healing cascade. Wound healing is a complex and multifaceted process that is vital for restoring tissue integrity after surgical procedures. It occurs in four distinct but overlapping phases, including hemostasis (immediate, minutes), inflammatory (hours to days), proliferation (days to weeks), and remodelling (weeks to months). Each phase is influenced by various factors such as optimal nutritional support, oxygenation, infection, patient-related issues, wound size, duration, and other factors [1].

Of these factors, nutritional support plays a crucial role, which has six classes: water, carbohydrates, fats, protein, vitamins, and minerals. Among these, vitamins play a pivotal role in soft tissue healing as they regulate cellular function, enhance collagen synthesis, modulate immune responses, and facilitate angiogenesis, all contributing to effective wound repair. Based on the solubility of vitamins, they are classified into fat-soluble (A, D, E, and K) and water-soluble (B complex and C), with a particular emphasis on vitamin D, which is well documented for its role in bone metabolism, immune function, and overall wound healing [2]. However, while its impact on bone health is widely recognized, its specific role in soft tissue healing remains less explored. Vitamin D deficiency has been associated with delayed wound closure, increased inflammation, and impaired epithelialization. In a systematic review by Smith and Hewlings [3], a strong association between low 25-hydroxyvitamin D levels and the occurrence of hard-to-heal wounds was identified. It has been estimated that 1 billion people worldwide have vitamin D deficiency or insufficiency.

Several researchers have emphasized the role of vitamin D in soft tissue healing, highlighting its potential to improve post-surgical recovery. A pilot investigation by Mameledzija et al. [4] explored the effect of vitamin D serum levels on third molar extraction outcomes, suggesting that adequate vitamin D may support better soft tissue healing after surgery.

This case report aims to explore the relationship between vitamin D deficiency and delayed wound healing following depigmentation, frenectomy, and crown lengthening procedures performed with electrosurgery, shedding light on its clinical implications and the need for adequate vitamin D levels to optimize healing outcomes.

## Case presentation

**Figure 1:**
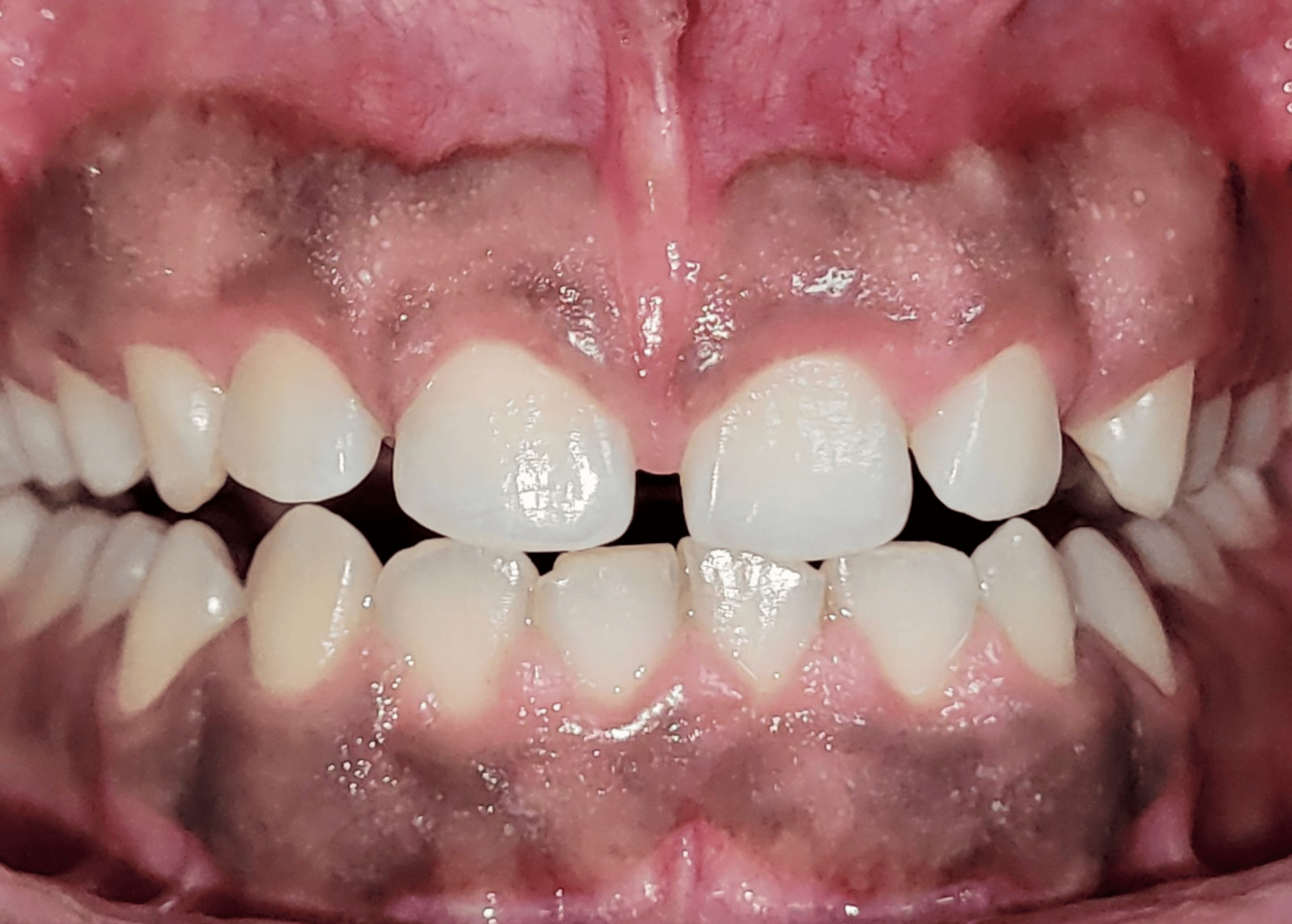
Preoperative image.

A 21-year-old male presented to the Department of Periodontology, Subharti Dental College and Hospital, Meerut in November 2023 with a chief complaint of unesthetic appearance and increasing gap between the front teeth for five months while smiling (Figure 1).

His medical history was non-significant, and he denied a history of adverse habits such as smoking and tobacco and alcohol consumption. The patient had no allergies to any medications. His recorded vitals were normal. Upon an intraoral examination, the patient had a band of melanin pigment along with short clinical height of crowns in both maxillary and mandibular arches (Figure 1). Additionally, a thick, aberrant maxillary labial frenum was noted, contributing to the midline diastema. The patient was educated about all possible treatment approaches, and, subsequently, informed consent was obtained from the patient.

**Figure 2:**
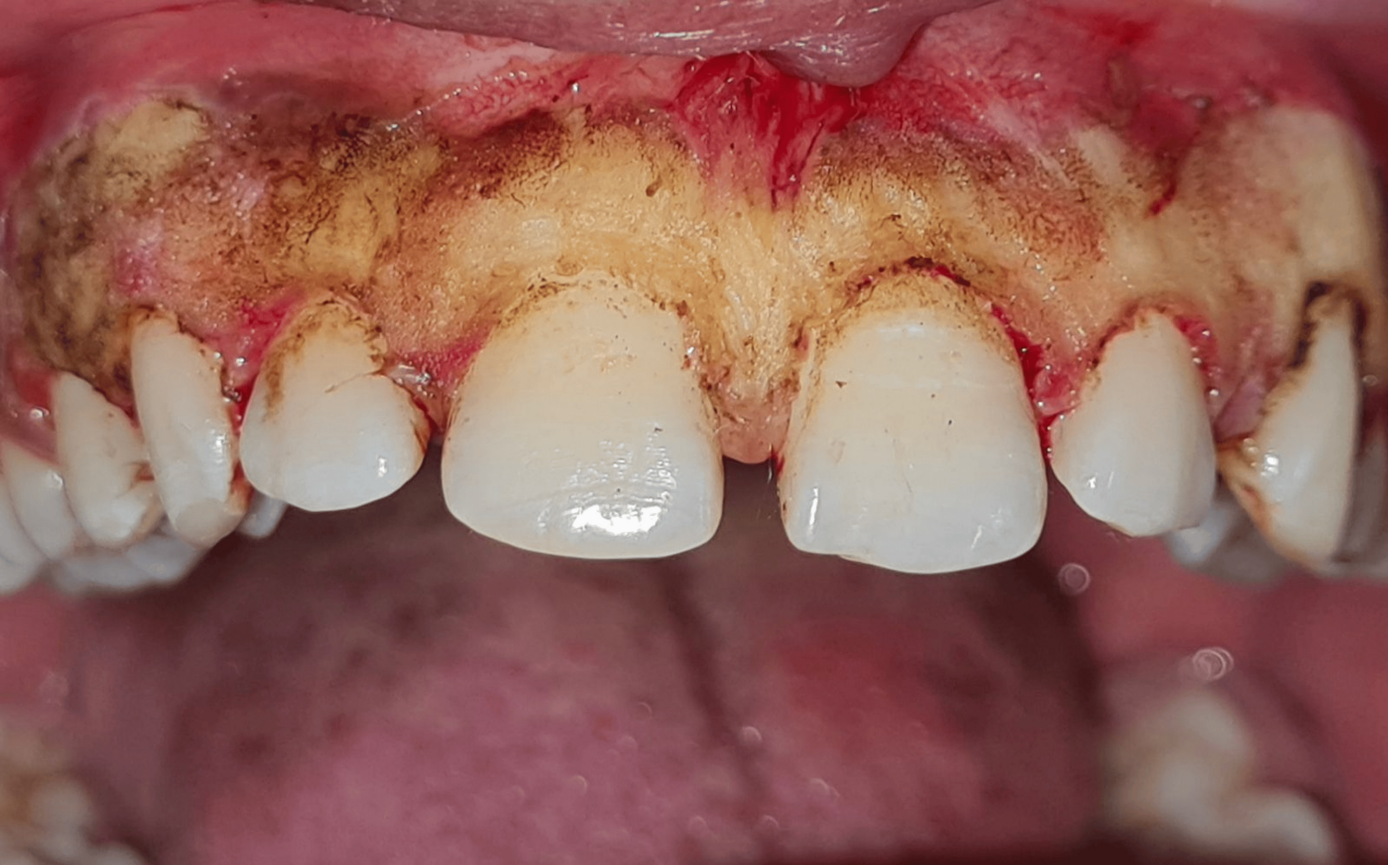
Immediate postoperative image.

The patient first underwent a crown lengthening procedure using an external bevel gingivectomy approach to expose more tooth structure, maintaining the gingival zenith, followed by frenectomy using a conventional scalpel technique to remove the aberrant frenum and reduce tension on the midline area. To address the pigmentation, an electrosurgical unit was used to remove the pigmented melanin band in the maxillary arch. The patient was called again after two days (48-hour follow-up), and the surgical site exhibited signs of delayed healing (Figure 2).

**Figure 3:**
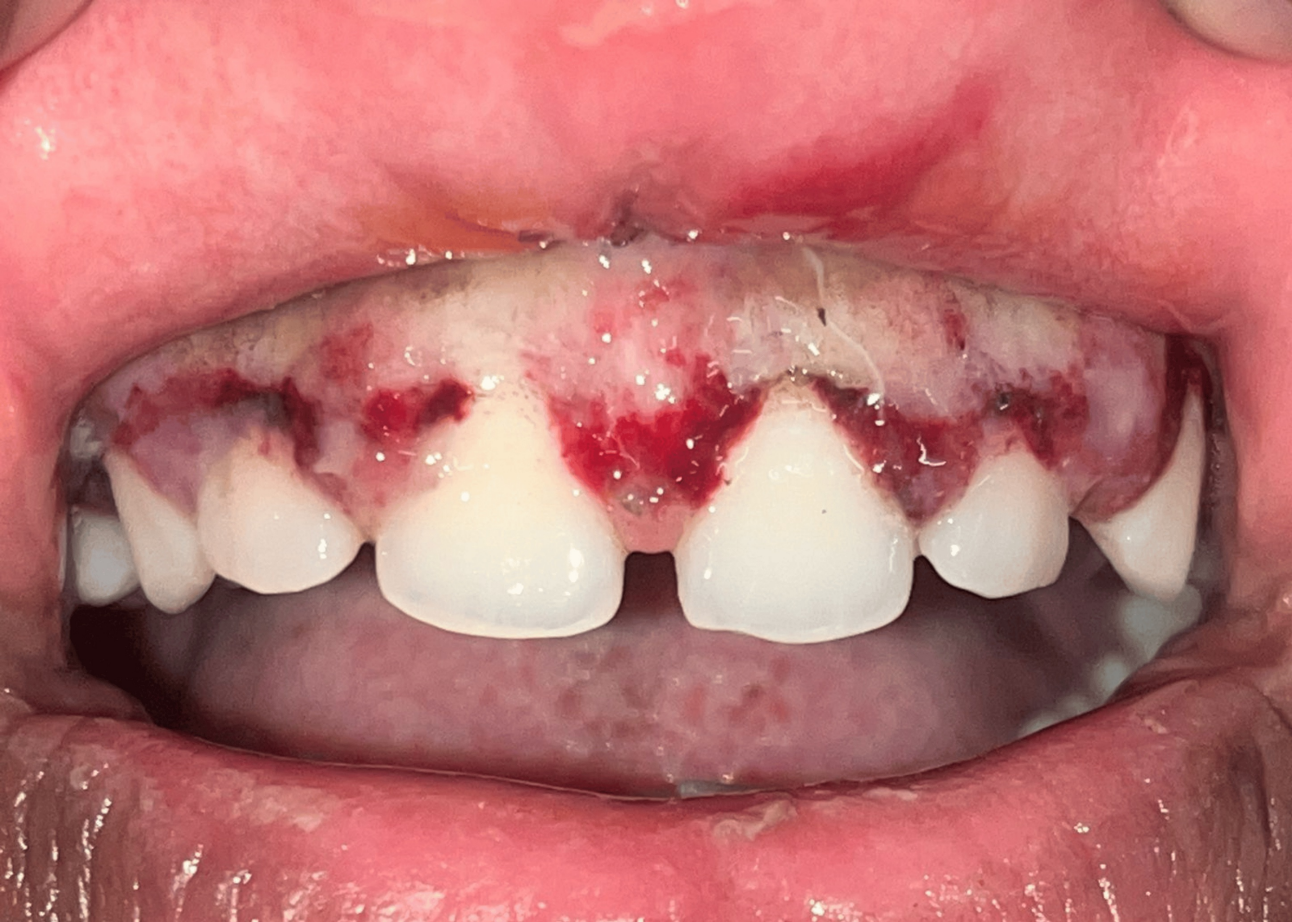
Seventh-day follow-up image.

**Figure 4:**
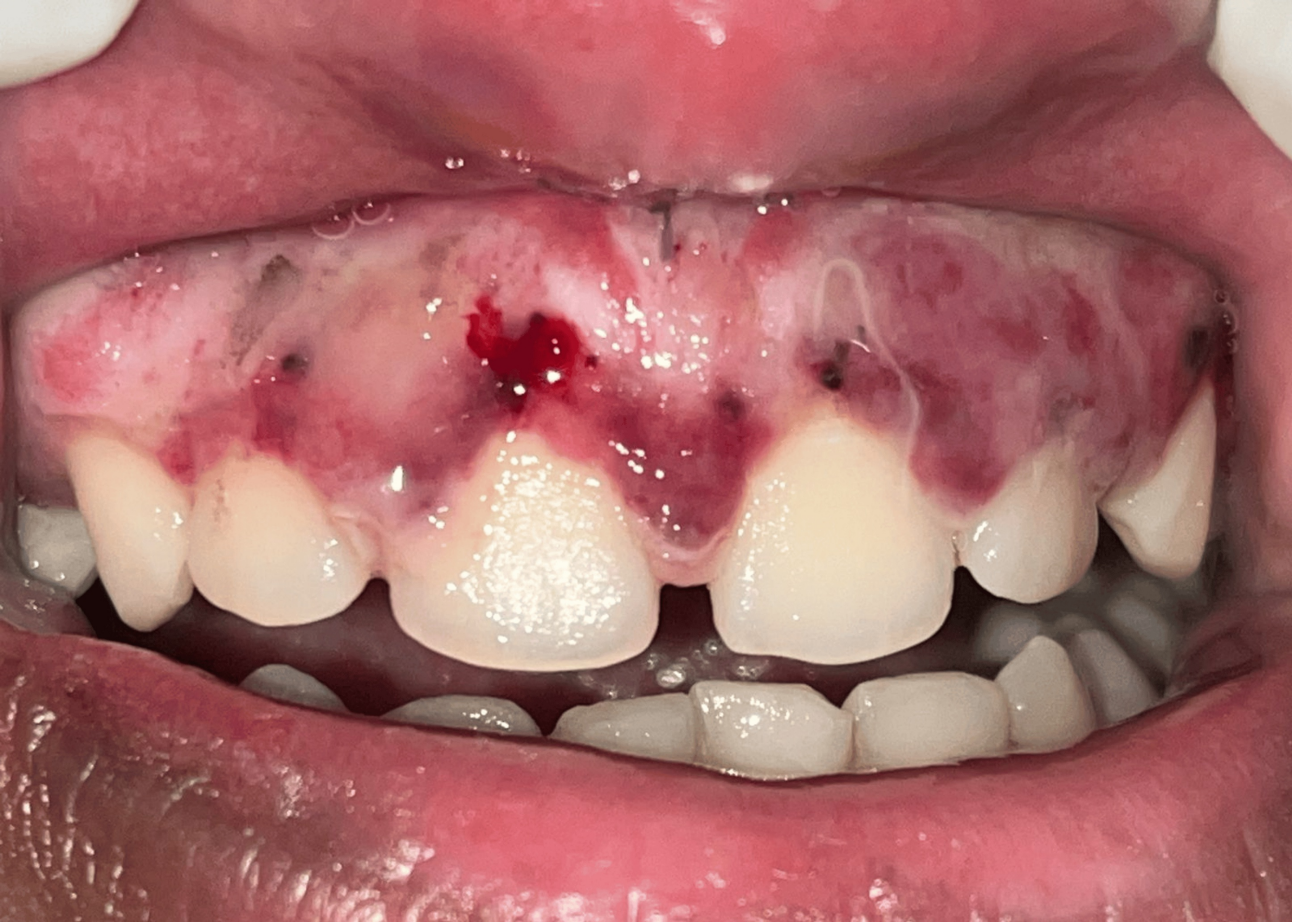
21st-day follow-up image.

**Figure 5:**
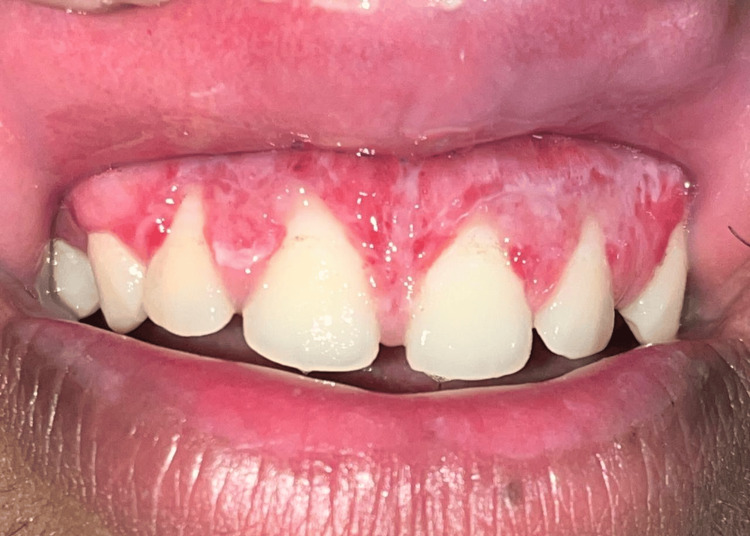
45th-day follow-up image.

At the seventh-day follow-up, persistent erythema, slight edema, and incomplete epithelialization were observed (Figure 3). By the third week, healing was still delayed along with persistent erythema and discomfort to the patient (Figures 4, 5).

**Figure 6:**
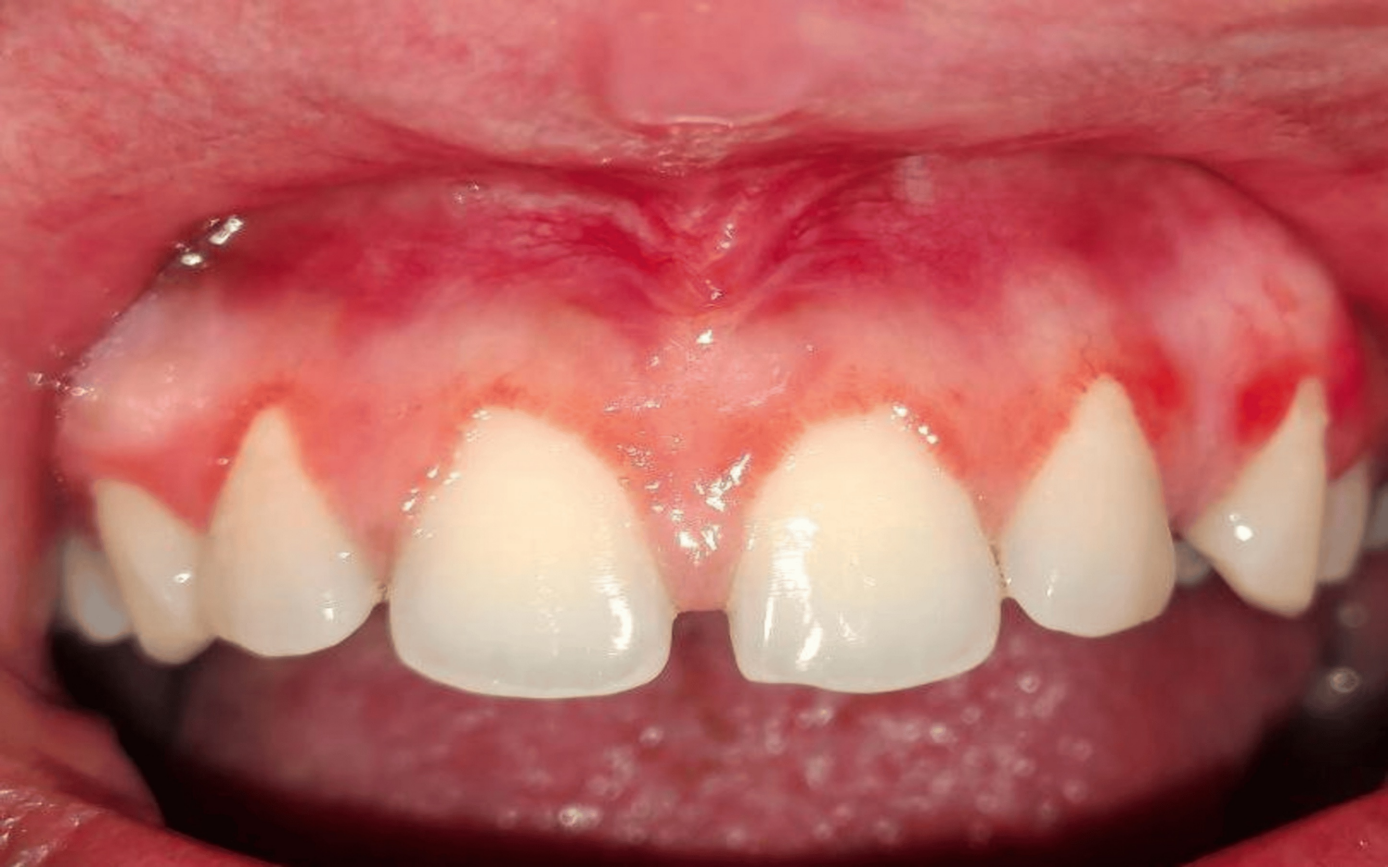
Third-month follow-up image.

**Figure 7:**
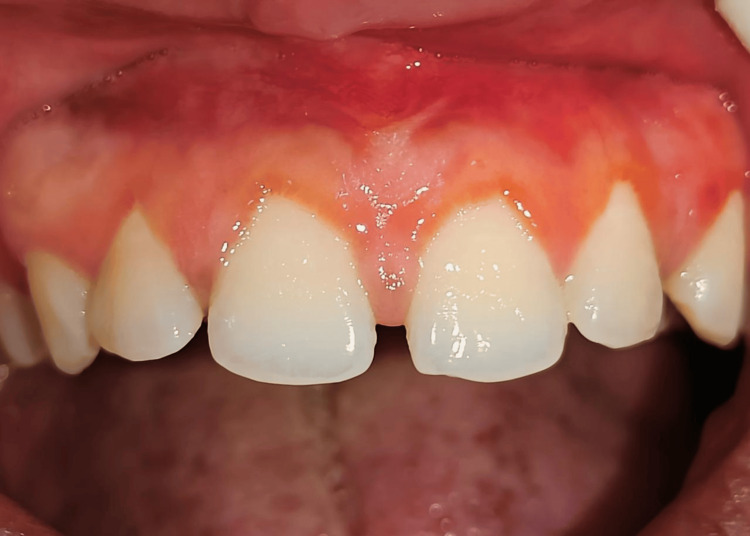
Fifth-month follow-up image.

Given the unexpected healing pattern, a blood investigation was advised. The results revealed a marked deficiency in serum 25-hydroxyvitamin D levels while calcium, phosphate, and other hematological parameters were within the normal range. The patient was referred to a physician and started on high-dose vitamin D3 supplementation (60,000 IU weekly for eight weeks), along with dietary advice. Within two weeks of initiating therapy, notable improvement in healing was observed, and by six weeks, the gingival tissues had completely healed with satisfactory aesthetic outcomes. The patient was kept on regular follow-ups at one, two, and five months (Figures 6, 7).

**Figure 8:**
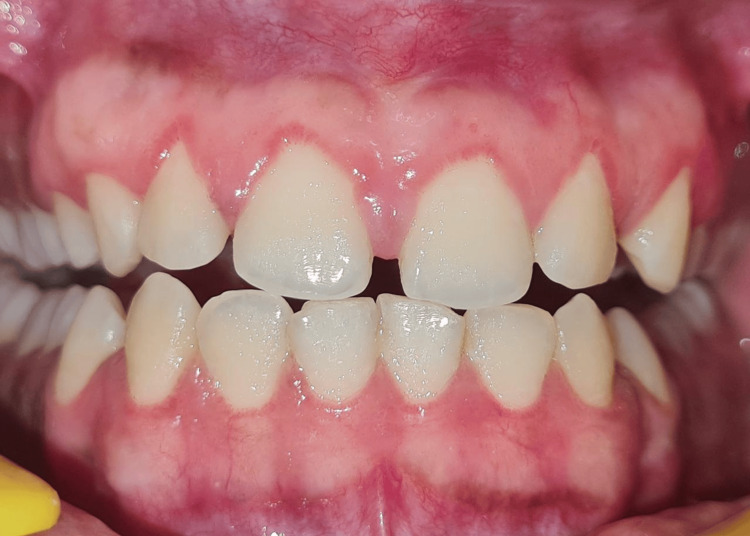
10th-month follow-up image.

Once the maxillary sextant was healed, depigmentation using electrosurgery was done in the mandibular arch. The patient was kept on maintenance vitamin D supplementation and remains under regular review. The patient was called again after 10 months, and satisfactory healing was achieved following vitamin D supplementation (Figure 8).

## Discussion

Delayed soft tissue healing refers to the prolonged or impaired recovery of the mucosal tissues within the oral cavity, which often results from a combination of local and systemic factors [5]. Contributing factors may include inadequate oral hygiene, systemic conditions such as diabetes mellitus or immunosuppression, tobacco use, nutritional deficiencies, certain medications (such as corticosteroids, chemotherapy agents, or bisphosphonates), and mechanical trauma from dental appliances.

Delayed wound healing in the oral cavity is uncommon due to its rich vascularity and regenerative potential. However, systemic deficiencies can significantly impair the healing process. This case report describes a rare instance of delayed gingival healing with a 10-month follow-up after periodontal surgery, which was ultimately attributed to vitamin D deficiency. This case highlights the importance of considering nutritional factors in cases of unexplained delayed tissue repair.

Vitamin D helps heal wounds by binding with the vitamin D receptor through calcitriol. It regulates the transcription downstream in different target cells by stimulating the production of mitogenic growth factors and receptors such as platelet-derived growth factor, epidermal growth factor receptor, and keratinocyte growth factor receptor [6].

This case illustrates the critical but often overlooked role of vitamin D in oral soft tissue repair, even in young and otherwise healthy individuals. Vitamin D facilitates angiogenesis, modulates inflammatory responses, and supports fibroblast function, all of which are essential for post-surgical healing. Its deficiency may present subtly and only become apparent in the context of surgical trauma or stress to the tissues. Vitamin D deficiency should be considered in the differential diagnosis of delayed oral wound healing, even in young, systemically healthy patients. Early identification and correction can significantly improve outcomes and prevent unnecessary interventions or misdiagnosis. This case highlights the role of a holistic and systemic approach in managing postoperative complications in aesthetic periodontal therapy.

The patient’s delayed healing, characterized by persistent erythema, edema, incomplete epithelialization, and mild discomfort, is consistent with findings from other studies. In a case series, Öztekin and Öztekin [7] reported delayed healing of recurrent aphthous stomatitis and traumatic ulcerative granuloma associated with vitamin D deficiency, with improvement following supplementation. Vitamin D influences wound healing through modulation of inflammatory responses, epithelial-mesenchymal transition, and extracellular matrix remodeling [8]. While the presented case focuses on oral soft tissue healing, systemic studies have underscored the broader implications of vitamin D deficiency on wound healing. A study by Zubair et al. [9] on diabetic foot ulcers found that vitamin D deficiency was associated with impaired wound healing, and supplementation improved healing outcomes.

## Conclusions

This case report contributes to the growing body of evidence suggesting that vitamin D deficiency can impair oral soft tissue healing, even in young, healthy individuals. It underscores the importance of considering vitamin D status in patients presenting with delayed wound healing, regardless of age or systemic health status. Further research is warranted to elucidate the underlying mechanisms and establish standardized guidelines for vitamin D supplementation.
